# Insecticide resistance in the sand fly, *Phlebotomus papatasi *from Khartoum State, Sudan

**DOI:** 10.1186/1756-3305-5-46

**Published:** 2012-03-07

**Authors:** Mo'awia Mukhtar Hassan, Sally Osman Widaa, Osman Mohieldin Osman, Mona Siddig Mohammed Numiary, Mihad Abdelaal Ibrahim, Hind Mohammed Abushama

**Affiliations:** 1 Department of Vector Biology and Biomedical Studies, Tropical Medicine Research Institute, National Centre for Research, P. O. Box 1304, Khartoum, Sudan; 2 Blue Nile National Institute for Communicable Diseases, University of Gezira, P. O. Box 101, Wad Madani, Sudan; 3 Department of Zoology, Faculty of Science, University of Khartoum, P. O. Box 321, Sudan

## Abstract

**Background:**

*Phlebotomus papatasi *the vector of cutaneous leishmaniasis (CL) is the most widely spread sand fly in Sudan. No data has previously been collected on insecticide susceptibility and/or resistance of this vector, and a first study to establish a baseline data is reported here.

**Methods:**

Sand flies were collected from Surogia village, (Khartoum State), Rahad Game Reserve (eastern Sudan) and White Nile area (Central Sudan) using light traps. Sand flies were reared in the Tropical Medicine Research Institute laboratory. The insecticide susceptibility status of first progeny (F1) of *P. papatasi *of each population was tested using WHO insecticide kits. Also, *P. papatasi *specimens from Surogia village and Rahad Game Reserve were assayed for activities of enzyme systems involved in insecticide resistance (acetylcholinesterase (AChE), non-specific carboxylesterases (EST), glutathione-S-transferases (GSTs) and cytochrome p450 monooxygenases (Cyt p450).

**Results:**

Populations of *P. papatasi *from White Nile and Rahad Game Reserve were sensitive to dichlorodiphenyltrichloroethane (DDT), permethrin, malathion, and propoxur. However, the *P. papatasi *population from Surogia village was sensitive to DDT and permethrin but highly resistant to malathion and propoxur. Furthermore, *P. papatasi *of Surogia village had significantly higher insecticide detoxification enzyme activity than of those of Rahad Game Reserve. The sand fly population in Surogia displayed high AChE activity and only three specimens had elevated levels for EST and GST.

**Conclusions:**

The study provided evidence for malathion and propoxur resistance in the sand fly population of Surogia village, which probably resulted from anti-malarial control activities carried out in the area during the past 50 years.

## Background

Leishmaniasis is a vector borne disease caused by a parasite of genus *Leishmania*. It is considered as a major public health problem, 88^th ^in the world causing morbidity and mortality [1]. The disease also causes serious economic loss and impedes socioeconomic development in many countries [1].

Leishmaniasis is an endemic disease spread over a wide geographical area in Sudan [2,3]. Cutaneous leishmaniasis (CL) occurs in a fluctuating pattern in the country mainly in the west, central and northern parts of Sudan [2]. Whereas, visceral leishmaniasis (VL) is endemic in Savannah areas extending from the Sudanese-Ethiopian border in the east to the banks of the White Nile in the west, and from Kassala in the North towards Blue Nile State to the south with scatter foci in Nuba Mountain and Darfur [3].

The sand fly *Phlebotomus papatasi *Scopoli (Diptera: Psychodidae) is the principal vector of *Leishmania major*, the causative agent of CL in Sudan [2]. This species is the most dominant CL vector in the area north of Khartoum where an epidemic of CL had occurred [2]. However, *P. papatasi *has a wide range of distribution in the country including many parts of the arid areas of Sudan [2,4].

Vector control using insecticide campaigns in many countries have been mainly applied against mosquitoes and so indirectly against other insect vectors. Improper use of insecticide towards vector control has led to the development of insecticide resistance in tropical countries. However, the development of insecticide resistance in the insect vector threatens the effectiveness of these control measures. The insecticide resistance in sand fly populations has been highlighted by Singh et al. [5] and Kishore et al. [6]. For example in India, studies revealed resistance of sand fly vectors to dichlorodiphenyltrichloroethane (DDT) [5,6]. Currently, studies on insecticide resistance have focused on biochemical and molecular bases, which serve as a means of identifying resistant genotypes in insect populations [7-9]. However, only a few reports worldwide have shown the potential involvement of enzymes in insecticide resistance to sand flies [7,10].

In Sudan, due to intensive use of insecticides by malaria control programmes and in agricultural practice, especially in northern parts of the country, sand flies may have developed resistance to these insecticides. However, to date no single study has been carried out to investigate insecticide resistance in sand flies in the country, although many studies have been conducted on resistance in the malaria vector [11,12]. Therefore, this study was carried out to establish baseline data on the susceptibility and/or resistance status of *P. papatasi *to different insecticides.

## Methods

### Sand fly collection sites

**Figure 1:**
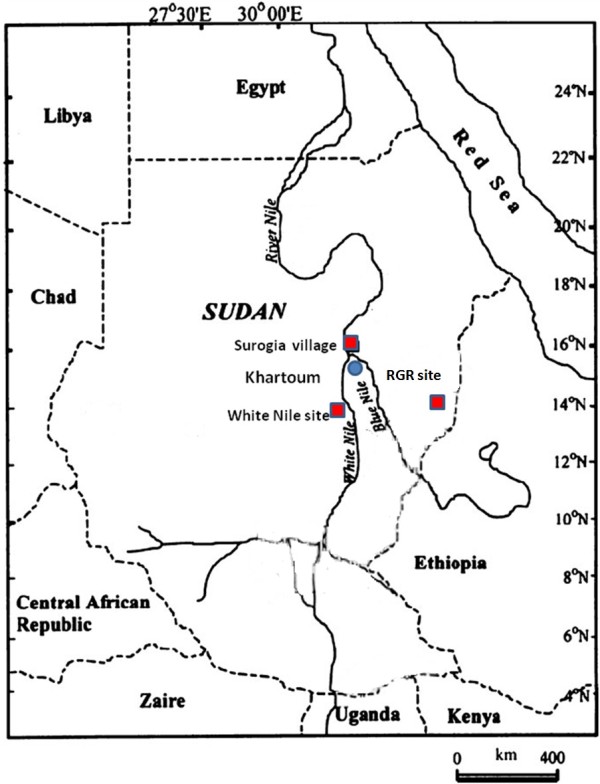
**A map of Sudan, showing the location of the study sites (Surogia village, White Nile area and Rahad Game Reserve)**.

In this study, sand flies were collected from three different geographical locations (Figure 1).

**Surogia village **(15°45' N, 32°15' E): the village is located on the eastern bank of the River Nile, about 30 km north of Khartoum. It is located in the endemic zone of CL [2]. The area is flat and covered by alluvium of still clay and sand deposited by the river. Surogia area experiences a climate of the semi-desert area with three distinct seasons, winter (November-February), summer (March-June), and autumn (July-October). Vegetation in the area is of the desert scrub type dominated by *Acacia *trees.

**White Nile area **(32°14' E, 14°72' N): the area is located on the western bank of the White Nile about 200 km south of Khartoum. The area is considered as a revived focus of (Visceral Leishmaniasis) (VL) [13]. Generally, the area is entirely located in the semi-desert dominated by desert scrub vegetation (i.e. *Acacia tortilis *and *Acacia mellifera*). This area is inhabited by the villagers and nomads. However, on a clay soil along the river, it was noticed an area of 7×30 km^2 ^occupied by low rainfall savannah vegetation characterized by *Acacia seyal/Balanities aegyptiaca *thicket.

**Rahad Game Reserve Camp **(35°11' E, 12°51' N): the area is located on the western bank of the Rahad river about 40 km from the main Galgeu Warden Camp (110 km from the Sudanese-Ethiopian border). The camp is adjacent to Bello village, about 1.5 km. The area is located in the endemic area of VL in eastern Sudan [14]. The ecology of the area was described by Elnaiem et al. [14]. The land is flat, but in many places it is interrupted by the seasonal rivers and tributaries and little ground surface water collection. The soil is mainly chromic vertisol (black cotton soil), with a few fractions of alluvial clays, sandy and silty soil known as "Azaza".

The climate of the area is tropical continental with an estimated annual rainfall of 1000 mm. The year is divided into dry (November-May), and rainy season (June-October). The vegetation of the area is dominated by savannah tree species such as *Acacia seyal*, *A. senegal*, *Balanites aegyptiaca *and *Ziziphus spina*-*christie*. The camp is inhabited by 6 people of the DNP Warden Camp. The DNP is protected by Sudanese Civil and Environmental law. Therefore, people are not allowed to carry out any activities (i.e. cultivation and malaria control programmes). However, the villagers near to the camp were allowed to graze cattle, sheep and goats.

### Collection and rearing of sand flies

Wild sand flies were collected from Surogia village and the White Nile area during March-April 2007 and April 2007 from Rahad Game Reserve using light traps set outdoors between 18:00 and 06:00 Hr. In each site sand flies were collected for 8 consecutive nights using 8 light traps where the traps were set at least 20 m from each other in an area of 150 m diameter.

In the laboratory, *Phlebotomus *species were sorted out from the captured sand flies and then transported to Khartoum. The sand flies of each of population were reared and maintained in the insectaries at the Tropical Medicine Research Institute (TMRI), Khartoum as described by Hassan et al. [15]. Briefly, in the laboratory, *Phlebotomus *sand flies were placed in a clean sand fly cage. Two guinea pigs (1-2 weeks old) were anesthetized (Thiopental at 20 mg/kg-intravenously) and introduced with sand flies in a sand flies cage for 30 minutes. After feeding, blood-engorged sand flies were individually put in oviposition vials lined at the bottom with gypsum (Calcium sulfate) material and covered with mesh. The vials containing females were then maintained at 28-30°C. After oviposition, females were removed from oviposition vials and were preserved individually in 70% alcohol for subsequent identification up to the species level by using a proper identification key constructed by Kirk and Lewis [16]. The gypsum material lining the oviposition pots was wetted with distilled water using long syringes. Emerging larvae were then fed with larval food composed of ground rabbit feces. The emerging adult females of *P. papatasi *(F1) were used for susceptibility and biochemical assays.

Animals used for sand fly feeding were obtained from an animal house of The Medicinal and Aromatic Plants Research Institute, National Centre for Research. When they were not used in feeding, the animals were kept individually in animal cages (12 sq ft with grids of 2.5 inches) and provided with food and water. Next morning, the animals were returned to the animal house.

The protocol used in this study was designed to follow the standard international guidelines for animal use in experimental research. Also, the protocol was approved by Human and Animal Research Ethics Committee of Tropical Medicine Research Institute, National Centre of Research and Research Ethical Committee of the Federal Ministry of Health (No: FMOH/RD/EC/64/08).

### Bioassay tests

Four insecticides (DDT, permethrin, propoxur and malathion) were used to determine the current status of insecticide resistance in the populations of *P. papatasi *from Surogia village, White Nile area and Rahad Game Reserve. The insecticides used were selected to represent different classes that have been used in Sudan. Standard WHO testing procedures were used to assess insecticide resistance using standard test kit tubes [17] under optimum conditions (temperature 26°C and 70 - 80% relative humidity).

DDT, permethrin, propoxur and malathion at concentrations 4%, 0.75%, 0.1% and 5% respectively, were used with an exposure time of 60 min. The doses of these insecticides were often used in the past as discrimination doses to separate susceptible from resistant phenotypic populations of sand flies [18]. The test kits were obtained from National Malaria and Leishmaniasis administration, Federal Ministry of Health, Sudan. The test was done in five replicates for each insecticide and one control using oil-treated paper. In each replicate, 20 unfed females were used. The numbers of knockdown and dead flies were recorded after 10, 20, 30, 40, 50 and 60 minutes of exposure. After one-hour exposure to insecticide impregnated papers, the knock-down and the surviving sand flies were transferred to clean holding tubes. The survivors were provided with a 30% sucrose solution on a piece of cotton. The final mortality was recorded after 24-hours.

### Biochemical analysis

Biochemical analysis was carried out as described by Hemingway [19] and modified by Surendran *et al*. [7]. Seventy-four and twenty adult fresh females of one day old, from Surogia village and Rahad Game Reserve populations respectively were individually homogenized in 200 μl of distilled water in a 1.5 ml eppendorff tube. The aliquots of supernatant from the homogenized flies were used in the four biochemical analyses described below (All readings taken for the replicates of each enzyme as mean optical density values).

### Total protein assay

Total protein of sand flies was analyzed as described by Bradford [20]. The assay was based on the observation of the maximum absorbance of an acidic solution of Coomassie Brilliant Blue G-250 (Sigma Aldrich, USA) shifts from 465 nm to 595 nm when binding to protein occurs. The reagent was prepared as described by Bradford [20]. Two replicates of 10 μl of each sand fly homogenate were placed in separate wells. 25 μl of NaOH was added to each sample and then 300 μl of Bradford reagent was mixed with sand flies homogenates in each well. The microtitre plate was incubated for 5 min and read at 570 nm using the ELISA microtitre plate reader. A series of concentrations (2.5 μg-3000 μg) of bovine serum albumin (Sigma Aldrich, USA) were used to prepare a typical standard curve for protein. The standard curve was used to convert the optical density for each sample to a concentration (in μg). The concentrations were used to calculate the activity of GST, α and β-naphthyl acetate.

### Acetylcholinesterase (AChE) activity

Two replicates, each of 25 μl of crude homogenate aliquot were transferred to a microtitre plate. Then, 145 μl of Triton phosphate buffer (1% (v/v) Triton X-100 in 0.1 M phosphate buffer pH 7.8) was added to each replicate. 10 μl of 0.01 M dithiobis 2-nitrobenzoic acid (DTNB) solution in 0.1 M phosphate buffer pH 7.0 and 25 μl of the substrate 0.01 M acetylthiocholine iodide (ASChI) (Sigma Aldrich, USA) was then added to one of the replicates to initiate the reaction. Also, 25 μl of ASChI containing 0.2% (v/v) of the inhibitor propoxur (0.1 M) was added to the second replicate. The kinetic reaction of the enzyme was continuously measured at 405 nm for 5 min in an ELISA reader. Then the inhibition percentage of AChE activity due to propoxur, as compared to uninhibited wells was calculated. The residual activity of more than 80% suggested homozygosity for an altered AChE whereas values between 60% and 80% suggested heterozygosity for sand flies [7].

### Non-specific esterase (ETS) activity

In this assay, two replicates of 20 μl of sand fly homogenate aliquot were transferred to separate wells in a microtitre plate. In the first replicate, 200 μl α-naphthyl acetate solution (100 μl of 30 mM α-naphthyl acetate in acetone diluted in 10 ml of 0.02 M phosphate buffer pH 7.2) was added and 200 μl β-naphthyl acetate solution (prepared as for α-naphthyl acetate) was added to the second replicate. The plate was incubated for 15 minutes at room temperature. Then 50 μl of Fast Blue Stain solution (150 mg Fast Blue in 15 ml distilled water), and 35 μl of 5% sodium lauryl sulphate (SDS) were added to each well. The first column in the microtitre plate containing all the reagents without sand fly homogenate was used as a negative control (Blank). Enzyme activity was read at 570 nm as an end point reading in an ELISA reader. Ranges of concentrations (2 μg-500 μg) α and β-naphthyl acetate (Sigma Aldrich, USA) solutions were used to setup standard curves to determine the concentrations of reaction products α and β-naphthyl acetate in μmol product min^-1^mg^-1 ^protein.

### Glutathione-S-transferase (GST) activity

GST activity was assayed by mixing 10 μl of homogenate aliquot with 200 μl of a substrate solution (95 parts of 10.5 mM reduced glutathione (Sigma Aldrich) in 100 mM phosphate buffer +5 parts of 63 mM 1-chloro 2,4-dinitrobenzene, CDNB, in methanol). The reaction rate was measured at 340 nm for 5 min using an ELISA reader. Enzyme activities were recorded as μmol product min^-1^mg^-1 ^protein. Then the remaining homogenate was centrifuged for 2 min at 10,000 g before aliquots of supernatant were removed for the following assays.

### Cytochrome p450 (Cyt p450) monooxygenases

10 μl of homogenate was mixed with 80 μl of potassium phosphate buffer (pH 7.2).

Then 200 μl of 6.3 mM tetramethyl benzidine (TMBZ) working solution (0.01 g TMBZ dissolved in 5 ml methanol and in 15 ml of sodium acetate buffer pH 5.0) and 25 μl of 3% (v/v) H_2_O_2 _solution in a microtitre plate well. After 2 hours incubation at room temperature, the plate was read at 630 nm as an end point [7] assay using an ELISA reader.

Values were compared with a standard curve of absorbance for known concentrations of cytochrome C and they were recorded as equivalent units of cytochrome p450 mg^-1 ^protein, for correcting the known haem content of cytochrome C and p450.

## Results

### Identification of *P. papatasi*

All females used for the establishment of the colonies of sand flies from the three geographic populations were identified morphologically as *P. papatasi*.

### Bioassay test

The results of the insecticide susceptibility tests are shown in table 1. According to the standard WHO [16] procedure, < 80% mortality 24-hours post exposure is considered as a strong indicator of resistant strains. In this experiment, discrimination doses previously used for sand flies and different exposure times for resistance and/or susceptibility were used. *Phlebotomus papatasi *from the Rahad Game Reserve and White Nile area populations were fully susceptible to permethrin, DDT, malathion and propoxur with a mortality level of 100% 24 hours post-exposure. The Population of *P. papatasi *from Surogia village was fully susceptible to permethrin and DDT with mortality rates of 100%, 24 hour post exposure, although the knockdown time value (at Cl 95%) obtained with permethrin was relatively high (KDT_95 _= 193.93 minutes) (Table 1). Malathion and propoxur resistance was detected in *P. papatasi *from Surogia village with the mortality rates of 19% and 9% for both insecticides respectively.

**Table 1:** Number and percentage of mortality and knockdown time values of female *Phlebotomus papatasi *from three different geographical areas in Sudan exposed to four different insecticides (WHO kits) after 24-hours exposure.

Sand fly population	Insecticide tested	No of tested (replicated)	Mortality (%) after 24 hours	KDT_95 _(minutes)
Surogia	Permethrin (0.75%)	100 (5)	100	193.93
	DDT (4%)	100 (5)	100	84.56
	Malathion (5%)	100 (5)	19	> 24 h
	Propoxur (0.1%)	100 (5)	9	> 24 h
	Control	100 (5)	1	
Rahad	Permethrin (0.75%)	100 (5)	100	20.17
Game	DDT (4%)	100 (5)	100	15.98
Reserve	Malathion (5%)	100 (5)	100	20.69
	Propoxur (0.1%)	100 (5)	100	22.02
	Control	100 (5)	2	
White Nile	Permethrin (0.75%)	100 (5)	100	20.16
area	DDT (4%)	100 (5)	100	15.98
	Malathion (5%)	100 (5)	100	22.02
	Propoxur (0.1%)	100 (5)	100	21.08
	Control	100 (5)	1	

KDT_95 _= Knockdown time of females (at Cl 95%)

### Biochemical tests

The procedures for the biochemical analysis of resistant sand flies [7] were used in this study. The level of enzymes of *P. papatasi *populations from Rahad Game Reserve and Surogia village were compared. Independent student t-tests revealed no significant differences (P > 0.05 for all enzymes) in the levels of α and β esterase enzymes (EST), glutathione S-transferase (GST) and cytochrome p450 monooxygenase (Cyt p450) between the sand flies of the two populations. However, the propoxur inhibited fractions of Acetylcholinesterase assay (AChE) enzymes showed a significant difference between *P. papatasi *of the two populations (t = 5.41; *P = *0.004).

**Figure 2:**
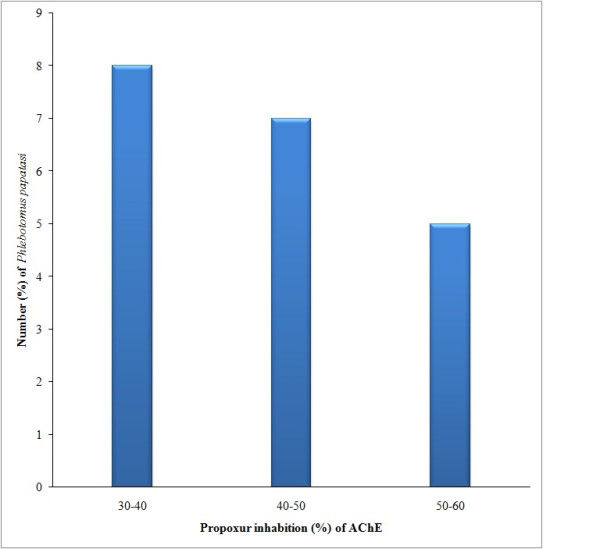
**Distribution of Acetylcholinesterase activity in *Phlebotomus papatasi *from Rahad Game Reserve (Dinder National Park; eastern Sudan) obtained by the biochemical test and used as a cut-of-point for susceptiblity/resistance**.

**Figure 3:**
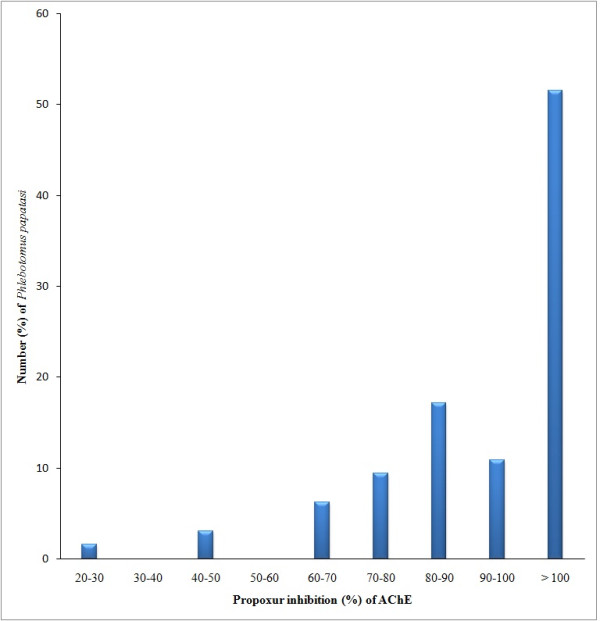
**Distribution of Acetylcholinesterase activity in *Phlebotomus papatasi *from Surogia village (Khartoum State, Sudan) obtained by the biochemical test**.

In this study, *P. papatasi *showed ranges of values for enzyme activities similar to those obtained for susceptible and resistant *P. argentipes *[7]. The levels of AChE activity, after incubation with 0.1 M Propoxur, equivalent to more than 80% of the activity without propoxur were used as indicator of insensitive AChE. Sand flies of Rahad Game Reserve showed an inhibition fraction ranging from 37.7-53.3% (Figure 2). Therefore, the specimens of Rahad Game Reserve were used to calculate the cut-off point for AChE inhibition fraction of the susceptible *P. papatasi *strain. Out of 64 specimens of *P*. *papatasi *assayed from Surogia village, more than 75% (79.7%) (n = 51) showed insensitive AChE (> 80% residual activity), suggesting resistance (homogenous resistance). Also, 50% of the population had an inhibited fraction of more than 100% (Figure 3).

However, 15.6% (n = 10) of the samples had 50-80% residual AChE activity (suggesting heterogeneous resistance) and 4.7% (n = 3) of the samples had < 50% residual AChE activity in the (suggesting homogenous susceptibility).

Similarly, specific EST activities of 0.02 ± 0.007 μmol min^-1 ^mg^-1 ^protein and 0.92 ± 0.08 007 μmol min^-1^mg^-1 ^protein were used as cut-off points for both susceptibility and resistance in *P. papatasi *respectively. In addition, GST specific activities of 0.42 ± 0.06 μmol min^-1^mg^-1 ^protein were used as cut-off points for susceptibility in *P.papatasi*. *Phlebotomus papatasi *of the Rahad Game Reserve had enzyme activities lower than those levels (EST; 0.0347 ± 0.003 and GST; 0.077 ± 0.002 μmole min^-1 ^mg^-1 ^protein). Almost, 95.9% (n = 71) of Surogia sand flies showed EST (0.048 ± 0.005 μmole min^-1 ^mg^-1 ^protein) and GST (0.097 ± 0.01 μmole min^-1 ^mg^-1 ^protein) activities at or below the elevated values, suggesting susceptibility. However, only three specimens out of 51 specimens with insensitive AChE had elevated values of EST (2.871 ± 0.49 μmole min^-1 ^mg^-1 ^protein) and GST (3.44 ± 0.5746 μmole min^-1 ^mg^-1 ^protein).

All *P. papatasi *assayed for Cyt p450 levels had values that fell within the susceptible range.

## Discussion

Insecticide resistance in vectors is a major public health problem especially in the tropical regions. There have been several reports of reductions in sand flies as a collateral benefit of malaria control programmes, although, these flies have developed resistance to insecticide, especially to DDT and less to other insecticides such as malathion and pyrethroids [21-26]. Therefore, it is of great value in view of control of sand flies and leishmaniasis to establish baseline data and to assess the extent of insecticide susceptibility and resistance in sand fly vectors, in order to design an effective control programme. No studies have previously been done to assess the susceptibility/resistance status of sand flies in the country, therefore, this study was carried out to establish baseline data for future control of sand fly vectors in Sudan.

The concentration of WHO insecticide kits used in this study had been used to test the susceptibility in sand flies and mosquitoes to insecticides [6,27-32]. The results of the susceptibility tests at 10, 20, 30, 40, 50 and 60 minutes of exposure indicated that *P*. *papatasi *populations from White Nile area and Rahad Game Reserve were fully susceptible to all insecticides used, whereas; the population of Surogia village was fully susceptible to DDT and permethrin but resistant to malathion and propoxur (Table 1).

These two populations were collected from areas where few or no agricultural practice or malaria control activities applied. The DNP is protected by Sudanese Civil and Environmental law against any human activities, therefore, no cultivation or malaria control programmes are allowed in this area. The collection sites in the White Nile area was from woodland (open area) about 5 km distance from villages and 3 km from farms. In contrast, the sand fly population of Surogia village was collected from the Khartoum area where intensive malaria control activities are regularly applied. Developing resistance to malathion and propoxur in the Surogia village population may be attributed to many years of insecticide usage for public health and agricultural purposes in northern Khartoum. No studies on insecticide resistance have been reported from our study sites in White Nile and Rahad River area, however, more recently, a study in Khartoum State revealed resistance in a field population of *Culex quinquefasciatus *to malathion, lambdacyhlothrin and permethrin [33].

The usage of insecticides for vector control started with benzine hexachloride (BHC) in Sudan back to 1950s [34]. But due to mosquito, insecticide resistance, BHC was replaced by DDT in 1965 and then to malathion in 1975 [34]. Moreover, due to resistance, malathion was also discontinued in 1979 and replaced by fenitrothion, and later in 1990 to deltamethrin, which is still considered to be effective. No reports are available on the agro-chemical use in Sudan, however, organochlorines and organophosphorus pesticides such as aldrin, chlordane, DDT, dieldrin, heptachlor, hexachlorobenzene and toxaphene have been used in large agricultural schemes e.g. Gezira agriculture scheme.

In this study, biochemical assays as described by Hemingway [19] and modified later by Surendran et al. [7] were used for the analysis of sand flies from two different geographical regions. The results revealed that only three individuals out of 74 sand flies had elevated EST, Cyt p459 and GST activities. This result might support the results shown by the WHO susceptibility test, which revealed that *P*. *papatasi *is highly susceptible to DDT and permethrin. It is known that these enzymes have been implicated in resistance in many insects of medical importance [35,36], as well as agricultural pests. Therefore, their association with resistance in these sand flies cannot be excluded although only three individuals had elevated level of EST, Cyt p459 and GST activities. For example the presence of few individuals with elevated GST enzymes there may likely be involved in residual resistance due to many years of DDT usage for the mosquito control programme during 1965-1975 [34]. The GSTs have been found to be involved in DDT resistance in sand flies [7] and mosquito species [37], as well as, in the OPs resistance [35].

Furthermore, significant difference in the level of AChE enzyme was observed between the sand fly populations of Rahad Game Reserve and Surogia village. However, AChE insensitivity in the presence of carbomate and propoxur was detected in the *P. papatasi *population (79.7%) from Surogia village. This result might suggest high levels of insecticide resistance in *P. papatasi *due to point mutations in the structural gene (*Ace*). Point mutation in the *ace *gene has been found to be associated with high levels of insecticide resistance especially, to OP-resistance and/or carbomates-resistance [38]. In our case, the high levels of resistance in *P. papatasi *could be due to back history of malathion and propoxur application in the anti-malaria vector control activities. In sand flies *Lutzomyia longipalpis*, insensitive AChE is caused by point mutations within a single gene (*AceI*), which led to significant resistance to insecticides [39].

In this study, a major problem was to establish a discriminating concentration or time for the susceptibility test and the values of resistant and susceptible sand flies for biochemical tests. Only a few studies have been carried out to establish discriminating doses for killing susceptible specimens of sand flies [18,26]. Also, a single study was carried out to establish cut off values of resistance and susceptibility in sand flies using biochemical analysis [7]. In this study, no resistant reference strain of *P. papatasi *was available, therefore we used discriminating doses and values of resistance and susceptible sand flies in susceptibility tests and biochemical analysis of those used for *P. papatasi *and *P. argentapis *[18,26,7]. However, the results obtained in this study suggest that populations of *P. papatasi *can be used in the future to establish cut-off points for susceptible strains in biochemical assays and discriminating concentrations and times for insecticides.

In this study, insecticide resistance in populations of *P. papatasi *was detected by two methods; WHO insecticide susceptibility tests and biochemical analysis. The insecticide test is often limited by availability of sand fly specimens whereas, the biochemical method is a technique used to determine the mechanism in individual insects; therefore only a small number of insects can be used. The caveat, however, is that, the resistance obtained by measuring enzyme activity, does not always correlate with resistance obtained by the susceptibility test, whereas the susceptibility test results may be an indicator for growing resistance problems, although it does not predict an operational failure of spraying programmes. Because resistance in insect vectors can be caused by various factors including method of application, the size of the insect population and insect genetics (frequencies of alleles involved in resistance) [40]. Moreover, insecticide resistance is likely to result in reduction of the vectorial capacity of the insect by affecting vector longevity, its infectiousness and change in its behaviour [41].

## Conclusions

This study has shown that the populations of *P. papatasi *in Surogia village were resistant to malathion and propoxur but susceptible to DDT and permethrin. However, before concluding that permethrin and DDT can be efficiently used to control sand flies, further studies are needed to determine effective gradients of DDT against sand flies. DDT has adverse human health and environmental effects of exposure. However, the World Health Organization and the Stockholm Convention 2001 have permitted its use only for indoor residual spraying (IRS) to control vector borne diseases [42,43]. Therefore, when using DDT in IRS programs, some operational factors should be considered, these are; adequate supply and distribution of quality DDT, appropriate storage and disposal of insecticides, effectively managed IRS programs, disease surveillance, and evaluation of any adverse effects of DDT to human health and the environment, supervision of trained sprayers, raise awareness among spraying personnel and targeted communities on issues relating to DDT use and preventing the dispersion of DDT into agriculture. Also, regular assessment and monitoring of the spread of vector resistance to DDT using conventional bioassays and biochemical assays have an important role in insecticide resistance management and to evaluate the future uses of insecticides for control strategies.

## Competing interests

The authors declare that they have no competing interests.

## Authors' contributions

MMH designed experiments, coordinated field and lab works, collected and analyzed data, drafted and revised the manuscript; SOW: participated in the field and the lab activities, helped in collected and analyzing the data and drafting the manuscript; OMO: participated in the field and the lab activities, and data analysis; MSMN: participated in the field and lab activities; MAI: contributed to field, lab activities and helped in data analysis; HMA: contributed to design lab analysis and revised the manuscript. All authors have read and agreed with the content of the submitted manuscript
